# Correlation between Vegetable and Fruit Intake and Cognitive Function in Older Adults: A Cross-Sectional Study in Chongqing, China

**DOI:** 10.3390/nu16183193

**Published:** 2024-09-21

**Authors:** Yingjiao Deng, Jiaxin Deng, Ke Jiang, Ya Shi, Ziling Feng, Rongxin Wu, Ailin Zhou, Zumin Shi, Yong Zhao

**Affiliations:** 1 School of Public Health, Chongqing Medical University, Chongqing 400016, China; 2023111615@stu.cqmu.edu.cn (Y.D.); 2022110613@stu.cqmu.edu.cn (J.D.); 2021110631@stu.cqmu.edu.cn (K.J.); 2023121694@stu.cqmu.edu.cn (Y.S.); 2022120844@stu.cqmu.edu.cn (R.W.); 2022120872@stu.cqmu.edu.cn (A.Z.); 2 Research Center for Medicine and Social Development, Chongqing Medical University, Chongqing 400016, China; 3 Research Center for Public Health Security, Chongqing Medical University, Chongqing 400016, China; 4 Nutrition Innovation Platform-Sichuan and Chongqing, School of Public Health, Chongqing Medical University, Chongqing 400016, China; 5 Department of Epidemiology and Health Statistics, Xiang Ya School of Public Health, Central South University, Changsha 410013, China; 236912065@csu.edu.cn; 6 Human Nutrition Department, College of Health Science, Qatar University, Doha 2713, Qatar; zumin@qu.edu.qa; 7 Chongqing Key Laboratory of Child Nutrition and Health, Children’s Hospital of Chongqing Medical University, Chongqing 400014, China

**Keywords:** food groups, older adults, cognitive function, MCI, Chongqing

## Abstract

Objective: To explore the correlation between different types of vegetable and fruit intake and cognitive function among the older adults in Chongqing, China, and to provide a scientific basis for developing efficient lifestyle interventions for the prevention of Mild Cognitive Impairment (MCI). Method: Approximately 728 older adults in urban and rural areas of Chongqing were surveyed using face-to-face questionnaires. Cognitive function was assessed with the Montreal Cognitive Assessment-Basic (MoCA-B) scale, and the vegetable and fruit intake groups were investigated with the Simple Food Frequency Counting Survey Scale. Binary logistic regression was used to explore the effect of the vegetable and fruit intake group on cognitive function. Subgroup analysis was used to demonstrate the robustness of the results. Result: Of the 728 participants in the study, 36.40% were likely to have MCI, which is higher than the national average for this condition. After adjusting for confounders, compared to the Q1 group, fruit and root vegetable intake was a protective factor for MCI, showing a dose–response relationship (*p* < 0.05). Only lower intake (Q2) of total vegetables, medium intake (Q2, Q3) of solanaceous vegetables, and medium–high intake (Q2, Q4) of fungi and algae was protective against MCI, whereas the leafy vegetables showed no relation to MCI. Apart from this, participants who were older, female, unmarried, non-smoking, and engaged in physical labor, and who had an average monthly income of less than 3000 RMB were more likely to suffer from cognitive impairment. Conclusion: This suggested that the fruit-intake groups and some vegetable-intake groups showed a protective effect on cognitive function, and might behave differently depending on their different intake and demographic characteristics. A sensible, healthy diet can help prevent MCI.

## 1. Introduction

Mild cognitive impairment (MCI) is a transitional state between normal aging and Alzheimer’s Disease (AD) [1], characterized primarily by the progressive loss of memory or other cognitive functions, that affects memory, thinking, orientation, comprehension, calculation, learning, language, and judgment in older adults [2], but does not affect the ability to perform activities of daily living and does not meet the diagnostic criteria for AD, i.e., the clinical prodromal stage of AD [3]. There are no effective therapeutic strategies to improve its development [4]. However, research has shown that healthy eating habits are an effective means of preventing or delaying the occurrence of MCI in older adults [5]. Therefore, identification of potential risk and protective factors leading to primary prevention is a key measure to effectively improve cognitive function and delay AD in older adults.

Dietary diversity, defined as the intake of different types of food or different foods within the same food group over a given period of time [6], is considered to be the key to a high-quality diet. Epidemiological studies have indicated that greater consumption of fruit and vegetables are related to better cognitive function that might be attributed to their high content of antioxidants, such as vitamin C, carotenoids, and other active ingredients [7]. A meta-analysis of 16 observational studies showed that increased intake of fruit and vegetables in older adults was associated with a 21% decrease in the prevalence of cognitive impairment [8]. A study found that higher consumption of foods such as vegetables and fruit was effective in reducing the risk of MCI [9,10], suggesting that intake of vegetables and fruit might be a protective factor against cognitive impairment in older adults. However, one study found a potential U-shaped association between vegetable consumption and MCI in older adults in low- and middle-income countries, with no significant difference between the highest and lowest vegetable consumption [11], while results from a study [12] in southern Italy showed no significant association between dietary habits and MCI in older adults. None of these results found an association between fruit and vegetable intake and MCI.

A report estimated that more than 40% of Chinese middle-aged adults failed to meet the vegetable and fruit recommendations of the World Health Organization [13]. In particular, fruit consumption was very low among older Chinese adults; the vast majority of adults aged 80 or older were reported to rarely or only occasionally consume fruit, although most of them ate vegetables almost every day [14]. Chongqing has a great deal of mountainous and hilly terrain, and it is one of the most humid areas in China. As well, the cuisine of Chongqing is characterized by spicy food [15]. In addition, a study showed that middle-aged and elderly people in Sichuan and Chongqing generally face insufficient intake of vegetables and fruit compared with other regions of the country, and this dietary imbalance is particularly prominent in rural areas, groups with lower literacy levels, and low-income groups [16]. Although previous studies have focused on the relationship between overall fruit and vegetable intake and MCI, as well as the effects of micronutrient intake and different dietary patterns on cognitive function [17,18], more in-depth research is needed on the specific effects of the type and quantity of fruit and vegetable intake on MCI. Currently, most of the evidence on the relationship between vegetable and fruit intake and cognitive function originates from studies in Western countries. For example, a German study [19] noted that the variety of vegetables and fruits consumed might be associated with improved cognitive function. In addition, a study [20] showed a dose–response relationship between high plant-based food intake and maintenance of cognitive function. However, in China, relatively few studies have been conducted on the association between the intake of specific types and amounts of fruits and vegetables and cognitive function in older adults. Therefore, this study investigates the relationship between fruit and vegetable intake and cognitive function in older adults in Chongqing and provides theoretical support for the development of effective lifestyle interventions to prevent MCI.

## 2. Materials and Methods

### Study Design and Sample

This study selected the Chongqing Municipality as the investigation area, and the official survey was carried out from 1 June to 1 November 2023. Undergraduate and college students in Chongqing served as investigators, carried out on-site surveys in households and communities, using convenience sampling to select the elderly in each community, and conducted face-to-face questionnaires in the field. In order to ensure the quality of the control study design, the investigators were strictly screened, and professional training was provided by the person in charge to ensure that each investigator possessed solid professional knowledge and investigation skills. At the same time, data integrity, logic, and consistency were strictly scrutinized, invalid questionnaires containing obvious outliers and other invalid questionnaires that did not meet the original screening criteria were eliminated, and qualified questionnaires were coded. Questionnaire entry training was provided to the entry clerks, including the “Questionnaire Star” operation specifications and notes for each question, to ensure accurate and efficient entry. Questionnaires were coded and assigned to each entry agent, and the automatic logical error checking, no-empty-item checking, and limit-response function of the Questionnaire Star were utilized to ensure the integrity of the entries. The sample size required for the study was calculated using the following sample size formula for cross-sectional surveys:

$$n=\frac{Z_{\alpha }^{2}\times pq}{d^{2}}$$

In the above sample size calculation formula, n is the required sample size, *p* represents the current prevalence of MCI in China, *q* = 1*p*, and let α = 0.05, then *Z_α_* = 1.96, with an allowable error of *d* = 0.2*p*, with reference to the currently recognized prevalence of MCI in China of 15.54% [3]. So, *p* = 0.15, *q* = 0.85, and *d* = 0.03, taking into account the effects of sampling error and invalid questionnaires and expanding the sample size based on the estimation by 10%; the final minimum sample size required is 575.

Inclusion Criteria: ① Residents over 60 years old in the Chongqing area ② with the ability to communicate independently who ③ voluntarily participated in this study and signed the informed consent form and ④ were able to cooperate with the dietary survey as well as the cognitive function test.

Exclusion Criteria: ① Refusal or inability to provide true and accurate information and ② severe hearing impairment, language impairment, cognitive impairment, or inability to communicate normally.

**Figure 1:**
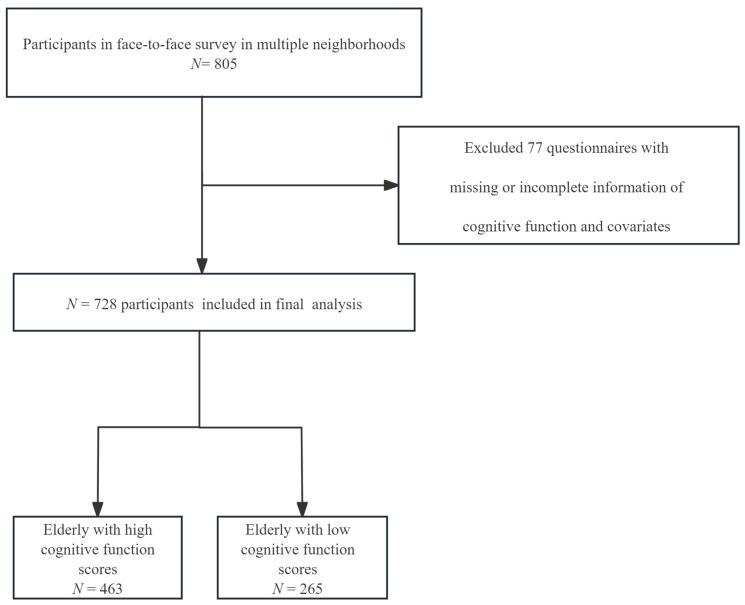
The flow chart of the inclusion of older adults.

Finally, 805 questionnaires were collected, and after excluding 77 invalid questionnaires, 728 questionnaires were included in the analysis (Figure 1).

## 3. Measures

### 3.1. Assessment of Cognitive Function

The Montreal Cognitive Assessment-Basic (MoCA-B) contains 10 questions [21]. The Montreal Cognitive Assessment Scale (MoCA) is a level A recommended assessment tool for the rapid screening of MCI in the 2018 Chinese Guidelines for the Diagnosis and Management of Dementia and Cognitive Impairment, which is more sensitive than the commonly used Mini mental state examination (MMSE). However, many of the questions in this scale are strongly influenced by education level. Considering that this study population will cover a large number of rural older adults, the new version of the MoCA (Montreal Cognitive Assessment-Basic, or MoCA-B), which was introduced, translated, and revised by Prof. Guo in 2015, was used in this study. This scale is used for MCI screening in illiterate and less-educated populations and has good reliability and validity in identifying MCI in Chinese older adults with different levels of literacy [22]. We categorized cognitive function scores into low and high scores based on the optimal threshold for identifying MCI in the MoCA-B scale identified in previous studies, while correcting for bias due to educational attainment. If a subject had ≤6 years of education and scored less than 18 points, they were considered to have a low cognitive function score, i.e., MCI may be present; if they had 7–12 years of education and scored less than 21 points, they were considered to have a low cognitive function score, i.e., MCI may be present; and if they had >12 years of education and scored at least 23 points, they were considered to have a low cognitive function score, i.e., possible MCI [23].

### 3.2. Assessment of Vegetable and Fruit Intake Frequency

A simplified food frequency method questionnaire [24], based on the elderly population in Chengdu, contained 24 common food groups. Some food entries were also modified in conjunction with the Dietary Guidelines for Chinese Residents (2022 edition), and the questioning method was improved. The Cronbach’s alpha coefficient of this scale was 0.768, indicating good reliability. The vegetables were categorized into leafy vegetables, solanaceous vegetables, root vegetables, and fungi and algae, and there was one fruit group. This was done by reviewing the number of times each food group was eaten by the respondents, which was then converted to an average daily intake (g), and the different food groups were categorized into Q1, Q2, Q3, and Q4, according to their low to high quartiles of intake.

### 3.3. Covariates

We reviewed the data to group the Body Mass Index (BMI) of different age groups of the elderly, and the criteria were as follows [25,26]: ① The appropriate range of BMI for the elderly (full age ≥ 80 years) in China is 22.0 kg/m^2^ ≤ BMI < 26.9 kg/m^2^; ② the appropriate range of BMI for the elderly aged 65 to 79 years is 20.0 kg/m^2^ ≤ BMI < 26.9 kg/m^2^; and ③ the appropriate range of BMI for the elderly aged 60 to 64 years is 18.5 kg/m^2^ ≤ BMI < 23.9 kg/m^2^. Marital status was categorized into two groups (married and other). Education level was divided into three categories (elementary and below; middle school; and high school and above) [27]. Pre-retirement job was categorized into three categories (mental labor: medical and health care industry, education-related industry, institutions, and commerce; physical labor: industry, agriculture, forestry, and fishery; and others) [28]. The values assigned to the relevant categorical variables are shown in Table 1.

## 4. Statistical Analysis

All statistical analyses of this study were conducted using Stata 18.0 software. All data were analyzed using a two-sided test, with *p* < 0.05 being statistically significant. Categorical variables such as sex in the basic socio-demographic information of the study subjects, BMI, etc., the percentage of high and low cognitive function scores, and exposure variables such as the total vegetable intake were used for descriptive statistics using frequency counts and percentages (n,%), and one-way analysis of variance was performed using the *χ*^2^ test. In the multifactorial analysis, binary logistic regression was used to explore the relationship between socio-demographic characteristic variables and cognitive function, as well as the effect of the vegetable and fruit intake groups on cognitive function, and the results were described using the Odds Ratio (OR) and its 95% Confidence Interval (CI). Also, the dose–response relationship between the vegetable and fruit intake groups and high or low cognitive function scores was examined by a linear trend test (*p* for trend). In order to effectively control for potential confounders, demographic characteristics found to be statistically different in the univariate analyses were adjusted, and three multivariate models were constructed. Model 1: No variable adjustment was made. Model 2: further adjusted for sex, age, and BMI. Model 3: further adjusted for residence, marital status, average monthly income, and pre-retirement job. In addition, subgroup analyses based on demographic characteristics were conducted for the vegetable and fruit intake groups that were significant in the multivariate model after adjusting for confounders. The *p*-value for the interaction was calculated by including the product term of the vegetable and fruit intake groups and the confounders in the regression model to further explore the interaction between the variables.

## 5. Result

### 5.1. Basic Demographic Characteristics, N = 728

As illustrated in Table 2, following the exclusion of the invalid sample, a total of 728 older adults were included in this study, of whom 10.4% were over 80 years of age (*p* < 0.05). With regard to sex distribution, there were more women (53.4%) than men (46.6%) (*p* = 0.017). The majority of the participants were married (76.9%), and 59.9% of the participants indicated that they had engaged in physical labour (*p* < 0.05). In terms of income, 49.3% of the participants indicated an income of 1000 to 3000 RMB (*p* = 0.006). Furthermore, 72.9% of the participants indicated that they had never smoked, and 70.5% had a healthy BMI (*p <* 0.05). Overall, there were statistically significant differences in age (year), sex, marital status, pre-retirement job, average monthly income, smoking, and BMI level (*p* < 0.05), while the education level and residence differences were not significant (*p* < 0.05).

### 5.2. Univariate Analysis of Variance of Intake of Different Food Groups in Groups with High and Low Cognitive Function Scores, N = 728

The univariate analysis of intake and high and low cognitive function scores in different food groups (Table 3), grouped according to quartiles of intake for each food group (Q1, Q2, Q3, and Q4), indicated that the differences in the intake of all vegetables, fruit, fungi and algae, leafy vegetables, solanaceous vegetables, and root vegetables were all statistically significant (*p* < 0.05). And all food intake subgroups showed the highest percentage of low cognitive function scores in the Q1 group (the low-intake group).

### 5.3. Binary Logistic Regression Analysis of Food Intake and Cognitive Scores

**Figure 2:**
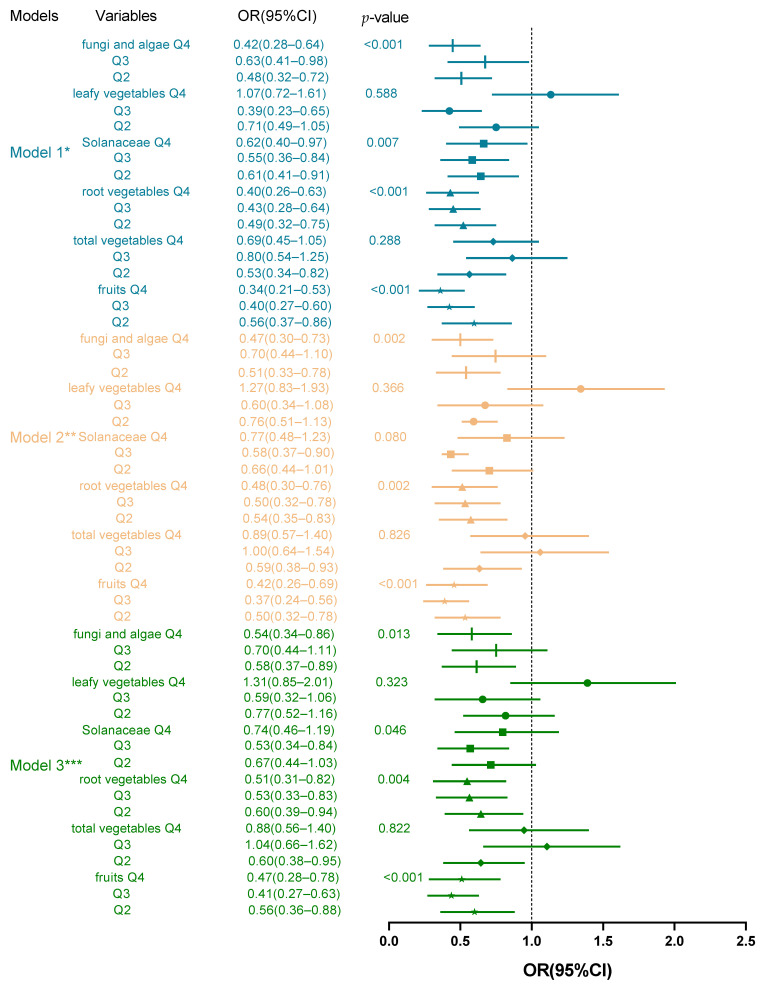
Binary logistic regression analysis of the effect of different food groups on high and low cognitive function scores. * Model 1: No variable adjustment was made. ** Model 2: further adjusted for sex, age, and BMI. *** Model 3: further adjusted for residence, marital status, average monthly income, and pre-retirement job.

Using binary logistic regression analysis (Figure 2), we found that medium to high intake levels (Q2, Q3, and Q4) of fruit and root vegetables were protective factors against cognitive impairment compared to the lowest-intake group (Q1), and after adjusting for confounders, the *p* for trend was still significant (*p* < 0.05), showing a dose–response relationship. The effects of fungi and algae intake on cognitive function differed before and after adjustment for confounders, with higher intake leading to higher cognitive function scores, but in Model 3, only the Q2 and Q4 levels showed protection for cognition, and group Q3 was not associated with cognitive function (OR: 0.70, 95% CI: 0.44–1.11). There were also different effects of solanaceous vegetable intake on cognitive function after adjusting the models. Specifically, Model 1 and Model 3 still showed better cognitive function with increasing intake (*p* < 0.05), but in Model 2, only the Q3 group showed high cognitive function scores (OR: 0.58, 95% CI: 0.37–0.90), while the other levels of intake (Q2 and Q4) showed irrelevant and non-significant *p* for trend (*p* = 0.08). In Model 3, only the Q3 group helped with cognitive impairment; intake at other levels failed to show a significant link. In leafy and total vegetable intake, there were no linear relationships; the *p* for trend was not significant (*p* > 0.05). However, in terms of total vegetable intake, the Q2 group showed a protective effect against cognitive impairment in all models. Additionally, before adjusting the models, the Q3 group of leafy vegetables showed to be a protective factor for cognitive function.

In general, after adjusting for all confounding factors within the food categories, higher intakes of fruit and root vegetables were associated with better cognitive function, a low total intake of vegetables, moderate intake of solanaceous vegetables, and moderately high intake of fungi and algae had a protective effect against MCI, and leafy vegetables did not appear to have an effect on MCI when dietary intake patterns were considered.

### 5.4. Subgroup Analysis of the Effects of Different Food Group Intake and Sociodemographic Characteristics on Cognitive Function Scores

We conducted subgroup analyses of food groups that showed a significant relationship with cognitive function scores after adjusting for confounding factors in logistic regression analysis, stratified by various sociodemographic characteristics. We selected demographic variables that were statistically significant in univariate analysis and calculated the *p* for the interaction between these demographic variables and the food groups.

### 5.5. Subgroup Analysis of the Effects of Fungi and Algae Intake and Socio-Demographic Associations on Cognitive Function Scores

Regarding fungi and algae intake (Table 4), we could see no interactions between fungi and algae intake and each demographic characteristic that were significant (*p* for interaction > 0.05). Yet, the effect of fungi and algae intake on high and low cognitive function scores remained a dose–response relationship in specific populations. In particular, among females, those in the 60~79 age group with a normal BMI, married people, those engaged in physical labor, those with a monthly income of less than 3000 RMB, and nonsmokers, compared to the low intake (Q1) group, the higher the intake of fungi and algae, the lower the possibility of cognitive impairment (*p* for trend < 0.05), whereas the above relationship was not proven in the rest of the population.

### 5.6. Subgroup Analysis of the Effects of Solanaceous Vegetable Intake and Socio-Demographic Associations on Cognitive Function Scores

Table 5 indicates that there was an interaction between sex, marital status, smoking, and the intake of solanaceous vegetables (*p* for interaction < 0.05), with a dose–response relationship observed among males, smokers, and married individuals. Furthermore, within specific populations, the impact of solanaceous vegetable intake on levels of cognitive function also demonstrated a dose–response relationship. Specifically, for individuals aged 60–79, with normal weight, earning less than 1000 RMB per month, and having been engaged in physical labor before retirement, the results revealed that higher solanaceous vegetable intake (Q2, Q3, and Q4) was associated with better cognitive function (*p* for trend < 0.05), whereas no such relationship was presented in other populations.

## 6. Discussion

Previous studies have shown that dietary factors are closely related to cognitive function [29]. So, in this study, we innovatively used the MoCA-B scale and the Simplified Food Frequency Approach Scale to investigate the current status of cognitive function and dietary intake in 728 older adults aged 60 years or older in Chongqing. And taking into account the relatively low literacy level of the population, we classified the different score lines according to their levels of education. In this research, binary logistic regression, subgroup analyses, and interaction analyses were used to identify the socio-demographic and dietary factors that were closely related to the cognitive function of older adults in Chongqing. There are few studies on the relationship between food groups and cognitive function in the elderly in Sichuan and Chongqing, and our study provides new regional insights into the field.

### 6.1. Basic Demographic Characteristics

The current study included 728 participants, of whom 36.4% had MCI. The prevalence was similar to that identified in a study in Shanghai [30], but higher than that found in other studies [31,32,33,34], probably because the majority of the elderly in this study had a low level of education, with 63.2% of them reporting primary school or less. The results analyzed in this study showed the increased risk of cognitive impairment in females, those above 80 years of age, unmarried older adults, those with a salary below 3000 RMB, those who had engaged in physical labour before retirement, non-smokers, and the thin elderly.

In line with prior empirical studies, our findings suggest that increasing age is associated with cognitive decline [35], which might be related to the aging of body functions and organs. With regard to sex differences, on one hand, it might be due to the small sample size of this study, and on the other hand, according to the Cognitive Reserve Hypothesis, for a long time, women had been more burdened with household chores, while men were more engaged in mental labour, resulting in sex differences between women and men, and overseas studies have also confirmed the above point of view [36]. A cross-sectional study in China found that widowed men were more likely to suffer from cognitive impairment [37] compared to those married; that is similar to our result. At the same time, several studies have confirmed that low income and low education levels are risk factors for cognitive decline [38,39,40]. This study found that people who were thin were more likely to develop MCI, which is similar to the findings of other studies that when BMI is below normal, the higher its value, the lower the risk of developing cognitive impairment [41]. Moreover, previous research shows that smoking could prevent or improve Parkinson’s disease and Alzheimer’s dementia [42].

### 6.2. Higher Fruit Intake Related to Lower Risk of MCI

Fruit is known to have positive effects on cognitive function. Most of the current research focuses mainly on the effect of total fruit and vegetable intake, whereas relatively few studies have explored the impact of fruit alone. Fruit is rich in polyphenols, antioxidant vitamins, and other phytochemicals, which can protect the brain and improve cognitive function [10,43,44,45]. A cohort study of community-dwelling older men in Hong Kong found that increased fruit variety intake was associated with a reduced risk of cognitive disability [46]. This was also confirmed in two longitudinal studies conducted by Huang et al. [47], wherein higher fruit intake was associated with better cognitive function and slower cognitive decline. But as a matter of fact, our fruit intake is generally insufficient at home. This is further supported by the data in this study, wherein the median fruit intake of the 728 study participants was only 53.57 g/day, which is far below the recommended intake of 200–350 g/day. This suggests that we need to pay attention to community health education on cognitive impairment, including MCI disease knowledge and dietary guidance [35] (Table S1).

### 6.3. Root Vegetable Intake Was Negatively Associated with MCI

Soluble dietary fibre is more abundant in root vegetables. While dietary fibre has beneficial effects on systolic blood pressure, body weight, stroke, and diabetes, on the other side, animal studies had shown that soluble dietary fibre improves neuroinflammation in Alzheimer’s dementia [48]. According to the study on cognitive function and influencing factors in Chinese older adults from 2005–2014, reduced vegetable intake was significantly associated with a higher prevalence of cognitive disability [49]. A study in Taiwan also supported the above view [50]. Another cohort study found there was a 23% lower risk of cognitive impairment in people who consumed more fruit and vegetables compared to those who consumed a lower variety of fruits and vegetables in low amounts [51]. The findings of Astrid et al. [52] indicate that there exist some differences among different variances of vegetables, and cabbage and root vegetables are associated with better cognitive function at baseline and/or smaller decline in cognitive domains. Consumption of root vegetables and mushrooms protects against the reduction of cognitive function [8] (Table S2).

### 6.4. The Effect of Fungi and Algae Consumption on MCI

Previous research suggests that mushroom consumption might have a preventive effect on cognitive impairment. And a Japanese cohort study found that the higher the mushroom consumption, the lower the risk of dementia [53]. These views were echoed by González-Domínguez et al. [54], who found a protective association between metabolites derived from the microbial metabolism of polyphenol-rich foods such as mushrooms and cognitive decline. As well, algae foods are rich in antioxidants that can keep the brain active and protect cognitive function. The study by Yang et al. [55] explored the association between the intake of edible mushrooms and algae and the risk of cognitive impairment in Chinese people aged 65 years and above. They noted that there was a 29% lower risk observed in those who ate fungi and algae daily, which can protect the brain from neurodegeneration by inhibiting the production of amyloid beta and exerting antioxidant effects, thereby improving cognitive function.

### 6.5. The Impact of Solanaceous Vegetables Intake on MCI

Our study found that before adjusting for confounding factors, the intake of solanaceous vegetables seemed to have a protective effect on cognitive function. However, after adjusting for confounding factors, the results showed that only the daily intake of 64.29 to 90 g of solanaceous vegetables could significantly reduce the risk of cognitive impairment. This suggests that there is, indeed, a suitable range of the protective effects of nut intake on cognitive function. Anthocyanin glycosides accumulate in the flowers, leaves, stems, and fruit of lycopene vegetables such as aubergines, tomatoes, chillies, and potatoes. And Yang et al. [56] showed that components such as anthocyanin glycosides in lycopene vegetables might positively affect cognitive function through their antioxidant and anti-inflammatory effects. Additionally, previous studies have shown that the diversity of vegetable intake is significantly associated with blood carotenoid and ascorbic acid concentrations, and the antioxidants in vegetables can prevent neurodegeneration by scavenging free radicals, thus improving cognitive deterioration [57].

### 6.6. The Effect of Leafy Green Intake on the Risk of MCI

A study conducted in the past examined the link between the key nutrients and bioactive compounds found in green leafy vegetables and the rate of cognitive decline in individuals. It was discovered that green leafy vegetables are particularly abundant in nutrients such as vitamin K (chlorophyll quinone), lutein, β-carotene, nitrate, folic acid, kaempferol, and α-tocopherol. The research suggests that consuming approximately one serving daily of green leafy vegetables and other foods abundant in these nutrients could potentially contribute to a slower rate of cognitive decline with aging [58]. In the research of Nooyens et al., a high consumption of leafy vegetables was correlated with superior episodic memory at the start and a reduced rate of cognitive decline [52]. Furthermore, other research indicates that a more diverse intake of vegetables is linked to higher cognitive function scores, implying that the range of vegetable types could be a protective element. Combinations of multiple types of vegetables might have additive or synergistic effects on physiology compared to nutrients from individual foods alone. Different vegetables contain different bioactive components, and the intake of multiple types of vegetables might allow for additive or synergistic effects on total antioxidant capacity and provide better protection of cognitive functions [57].

### 6.7. Strengths and Limitations

Regarding the link between diet and cognitive function, current research focuses more on Alzheimer’s and dementia and less on cognitive impairment. Our study categorized fruit and vegetables to explore the effects of food groups such as fruit, leafy vegetables, and root vegetables on MCI separately. However, this study has some limitations. First, the semi-quantitative food frequency method survey scale used in this study was based on the recommended amount information in the Dietary Guidelines for the Elderly in China (2022) to set the standardised intake of each food item per visit by age group and sex, which might not fully and accurately reflect the intake of each survey participant. In addition, the intake of nutritional supplements of the survey respondents was not collected. Further, the study was a cross-sectional study and was unable to establish the exact causal relationship between food groups and dietary nutrients and cognitive function, which needs to be explored more comprehensively in subsequent cohort or intervention studies. The causes of MCI are diverse, with Parkinson’s, cardiovascular and cerebrovascular diseases, and Alzheimer’s disease all being possible. This study did not analyze the medical history data of the elderly people in Chongqing and did not explore the causes of MCI in the participants who suffered from it, which can be used as a reference to carry out in-depth analyses for subsequent studies. The categories of MCI can also be analyzed in further studies.

## 7. Conclusions

There might be worse cognitive function in those who are older, female, not married, engaged in physical labour, earning less than 3000 RMB per month, and non-smoking. Fruit and root vegetable intake might protect people from MCI, showing a dose–response relationship that remained unchanged after adjusting for confounders in our study. Only the low total intake of vegetables, medium intake of solanaceous vegetables, and medium–high intake of fungi and algae were protective against MCI, whereas leafy vegetable intake showed no relation to MCI.

## Figures and Tables

**Table 1:** Variable assignments.

Variables	Variable Assignment Description
Total intake of vegetables	Q1 (<145.85 g/d), Q2 (145.85~224.64 g/d), Q3 (224.64~335.35 g/d), Q4 (≥335.35 g/d)
Intake of fruit	Q1 (<21.43 g/d), Q2 (21.43~53.57 g/d), Q3 (53.57~115 g/d), Q4 (≥115 g/d)
Intake of fungi and algae	Q1 (<2 g/d), Q2 (2~4 g/d), Q3 (4~8.57 g/d), Q4 (≥8.57 g/d)
Intake of leafy vegetables	Q1 (<82.14 g/d), Q2 (82.14~115 g/d), Q3 (115~150 g/d), Q4 (≥150 g/d)
Intake of solanaceous vegetables	Q1 (<25.71 g/d), Q2 (25.71~64.29 g/d), Q3 (64.29~90 g/d), Q4 (≥90 g/d)
Intake of root vegetables	Q1 (<14.64 g/d), Q2 (14.64~25.71 g/d), Q3 (25.71~82.14 g/d), Q4 (≥82.14 g/d)
Age (year)	0 = “60~79”, 1 = “≥80”
Sex	0 = Male, 1 = Female
BMI	0 = Underweight, 1 = Normal weight, 2 = Overweight/Obesity
Residence	0 = Urban, 1 = Rural
Marital status	0 = Married, 1 = Other
Education level	0 = Elementary and below, 1 = Middle School, 2 = High School and above
Pre-retirement job	0 = Mental labor, 1 = Physical labor, 2 = Other
Average monthly income	0 = “<1000 RMB”, 1 = “1000 RMB–3000 RMB”, 2 = “>3000 RMB”
Cognitive function score	0 = High, 1 = Low (MCI)

**Table 2:** Basic demographic characteristics (*N* = 728).

Variables	Cognitive Function Scores		*N* (%)	Chi-Square	*p*-Value
	High	Low (MCI)			
Age (year)				34.556	<0.001 **
60~79	438 (94.6%)	214(80.8%)	652(89.6%)		
≥80	25 (5.4%)	51 (19.2%)	76(10.4%)		
Sex				5.655	0.017 *
Male	231 (49.9%)	108 (40.8%)	339 (46.6%)		
Female	232 (50.1%)	157 (59.2%)	389 (53.4%)		
Marital status				13.165	<0.001 **
Married	376 (81.2%)	184 (69.4%)	560 (76.9%)		
Other	87 (18.8%)	81 (30.6%)	168 (23.1%)		
Education level				4.310	0.120
Elementary and below	284 (61.3%)	176 (66.4%)	460 (63.2%)		
Middle school	122 (26.3%)	69 (26.0%)	191 (26.2%)		
High school and above	57 (12.3%)	20 (7.5%)	77 (10.6%)		
Residence				1.183	0.280
Urban	317 (68.5%)	171 (64.5%)	488 (67.0%)		
Rural	146 (31.5%)	94 (35.5%)	240 (33.0%)		
Pre-retirement job				17.250	<0.001 **
Mental labor	107 (23.1%)	38 (14.3%)	145 (19.9%)		
Physical labor	251 (54.2%)	185 (69.8%)	436 (59.9%)		
Other	105 (22.7%)	42 (15.8%)	147 (20.2%)		
Average monthly income				10.208	0.006 *
<1000 RMB	121 (26.1%)	90 (34.0%)	211 (29.0%)		
1000 RMB–3000 RMB	226 (48.8%)	133 (50.2%)	359 (49.3%)		
>3000 RMB	116 (25.1%)	42 (15.8%)	158 (21.7%)		
Smoking				9.429	0.002 *
Non-smoker	320 (69.1%)	211 (79.6%)	531 (72.9%)		
Smoker	143 (30.9%)	54 (20.4%)	197 (27.1%)		
BMI				10.701	0.005 *
Normal weight	337 (72.8%)	176 (66.4%)	513 (70.5%)		
Underweight	59 (12.7%)	58 (21.9%)	117 (16.1%)		
Overweight/obesity	67 (14.5%)	31 (11.7%)	98 (13.5%)		

* *p* < 0.05, ** *p* < 0.001.

**Table 3:** Univariate analysis of variance of intake of different food groups in groups with high and low cognitive function scores (*N* = 728).

Variables	Cognitive Function Scores		*N* (%)	Chi-Square	*p*-Value
	High	Low (MCI)			
Total intake of vegetables				17.563	0.029 *
Q1	103 (22.2%)	79 (29.8%)	182 (25.0%)		
Q2	130 (28.1%)	53 (20.0%)	183 (25.1%)		
Q3	112 (24.2%)	71 (26.8%)	183 (25.1%)		
Q4	118 (25.5%)	62 (23.4%)	180 (24.7%)		
Intake of fruit				11.115	<0.001 **
Q1	100 (21.6%)	104 (39.2%)	204 (28.0%)		
Q2	101 (21.8%)	59 (22.3%)	160 (22.0%)		
Q3	156 (33.7%)	65 (24.5%)	221 (30.4%)		
Q4	106 (22.9%)	37 (14.0%)	143 (19.6%)		
Intake of fungi and algae				23.362	<0.001 **
Q1	139 (30.0%)	123 (46.4%)	262 (36.0%)		
Q2	121 (26.1%)	51 (19.2%)	172 (23.6%)		
Q3	82 (17.7%)	46 (17.4%)	128 (17.6%)		
Q4	121 (26.1%)	45 (17.0%)	166 (22.8%)		
Intake of leafy vegetables				22.174	<0.001 **
Q1	146 (31.5%)	103 (38.9%)	249(34.2%)		
Q2	135 (29.2%)	68 (25.7%)	203(27.9%)		
Q3	91 (19.7%)	25 (9.4%)	116(15.9%)		
Q4	91 (19.7%)	69 (26.0%)	160(22.0%)		
Intake of solanaceous vegetables				8.985	0.011 *
Q1	156 (33.7%)	122 (46.0%)	278(38.2%)		
Q2	113 (24.4%)	54 (20.4%)	167(22.9%)		
Q3	106 (22.9%)	46 (17.4%)	152(20.9%)		
Q4	88 (19.0%)	43 (16.2%)	131(18.0%)		
Intake of root vegetables				30.254	<0.001 **
Q1	89 (19.2%)	93 (35.1%)	182(25.0%)		
Q2	121 (26.1%)	62 (23.4%)	183(25.1%)		
Q3	144 (31.1%)	64 (24.2%)	208(28.6%)		
Q4	109 (23.5%)	46 (17.4%)	155(21.3%)		

* *p* < 0.05, ** *p* < 0.001.

**Table 4:** Subgroup analysis of the effects of fungi and algae intake and socio-demographic associations on cognitive function scores.

	Intake of Fungi and Algae				*p* for Trend	*p* for Interaction
	Q1 (<2 g/d)	Q2 (2~4 g/d)	Q3 (4~8.57 g/d)	Q4 (≥8.57 g/d)		
Age						0.752
60–79	ref	0.49 (0.31–0.75)	0.61 (0.38–0.98)	0.40 (0.25–0.63)	<0.001 **	
≥80	ref	0.56 (0.15–2.02)	1.25 (0.28–5.60)	0.69 (0.20–2.43)	0.727	
Sex						0.893
Male	ref	0.51 (0.27–0.97)	0.79 (0.40–1.56)	0.47 (0.25–0.88)	0.060	
Female	ref	0.50 (0.29–0.86)	0.57 (0.32–1.02)	0.44 (0.24–0.83)	0.004 *	
BMI						0.726
Normal weight	ref	0.49 (0.30–0.81)	0.74 (0.45–1.22)	0.38 (0.23–0.64)	0.001 **	
Underweight	ref	0.34 (0.14–0.81)	0.37 (0.11–1.30)	0.53 (0.17–1.60)	0.119	
Overweight/Obesity	ref	0.60 (0.18–2.07)	0.62 (0.16–2.34)	0.76 (0.27–2.16)	0.598	
Marital status						0.552
Married	ref	0.58 (0.36–0.92)	0.63 (0.38–1.07)	0.44 (0.27–0.72)	0.001 **	
Other	ref	0.29 (0.12–0.71)	0.63 (0.28–1.45)	0.45 (0.19–1.07)	0.080	
Pre-retirement job						0.996
Mental labor	ref	0.65 (0.24–1.74)	0.61 (0.19–1.95)	0.46 (0.17–1.26)	0.146	
Physical labor	ref	0.49 (0.29–0.83)	0.64 (0.38–1.09)	0.46 (0.27–0.78)	0.005 *	
Other	ref	0.42 (0.16–1.07)	0.56 (0.18–1.76)	0.38 (0.13–1.06)	0.057	
Average monthly income						0.879
<1000 RMB	ref	0.54 (0.26–1.12)	0.82 (0.39–1.71)	0.36 (0.15–0.90)	0.047 *	
1000 RMB–3000 RMB	ref	0.42 (0.24–0.77)	0.59 (0.31–1.11)	0.49 (0.27–0.87)	0.015 *	
>3000 RMB	ref	0.86 (0.29–2.55)	0.78 (0.23–2.61)	0.63 (0.21–1.88)	0.368	
Smoking						0.985
Non-smoker	ref	0.49 (0.31–0.77)	0.64 (0.39–1.07)	0.44 (0.27–0.72)	0.001 **	
Smoker	ref	0.47 (0.19–1.20)	0.74 (0.31–1.80)	0.50 (0.22–1.13)	0.170	

* *p* < 0.05, ** *p* < 0.001.

**Table 5:** Subgroup analysis of the effects of solanaceous vegetable intake and socio-demographic associations on cognitive function scores.

	Intake of Solanaceous Vegetables				*p* for Trend	*p* for Interaction
	Q1 (<25.71 g/d)	Q2 (25.71~64.29 g/d)	Q3 (64.29~90 g/d)	Q4 (≥90 g/d)		
Age						0.071
60–79	ref	0.55 (0.35–0.85)	0.44 (0.28–0.71)	0.66 (0.42–1.04)	0.009 *	
≥80	ref	1.86 (0.49–7.00)	2.17 (0.59–8.02)	0.62 (0.13–2.91)	0.816	
Sex						0.029 *
Male	ref	0.30 (0.15–0.60)	0.38 (0.20–0.72)	0.41 (0.22–0.76)	0.010 *	
Female	ref	0.97 (0.58–1.63)	0.73 (0.42–1.30)	1.13 (0.55–2.30)	0.714	
BMI						0.110
Normal weight	ref	0.66 (0.41–1.07)	0.48 (0.29–0.80)	0.55 (0.33–0.92)	0.005 *	
Under weight	ref	0.59 (0.21–1.65)	1.66 (0.64–4.32)	1.04 (0.34–3.18)	0.556	
Overweight /Obesity	ref	0.47 (0.16–1.37)	0.09 (0.01–0.75)	0.90 (0.27–3.02)	0.241	
Marriage						0.045 *
Married	ref	0.47 (0.29–0.76)	0.60 (0.37–0.98)	0.53 (0.32–0.88)	0.009 *	
Other	ref	1.36 (0.60–3.08)	0.43 (0.18–1.00)	1.04 (0.43–2.49)	0.412	
Pre-retirement job						0.087
Mental labor	ref	1.51 (0.56–4.06)	1.26 (0.48–3.32)	1.26 (0.41–3.83)	0.635	
Physical labor	ref	0.38 (0.22–0.64)	0.40 (0.24–0.67)	0.51 (0.30–0.88)	0.002 *	
Other	ref	1.09 (0.47–2.52)	0.53 (0.15–1.83)	0.48 (0.16–1.49)	0.138	
Average monthly income						0.151
<1000 RMB	ref	0.73(0.37–1.44)	0.25 (0.10–0.58)	0.38 (0.16–0.91)	0.002 *	
1000 RMB–3000 RMB	ref	0.46 (0.25–0.87)	0.76 (0.43–1.34)	0.79 (0.44–1.42)	0.459	
>3000 RMB	ref	0.67 (0.27–1.67)	0.65 (0.24–1.78)	0.61 (0.21–1.76)	0.292	
Smoking						0.029 *
Non-smoker	ref	0.68 (0.43–1.06)	0.81 (0.50–1.31)	0.90 (0.53–1.51)	0.525	
Smoker	ref	0.42 (0.17–1.03)	0.19 (0.07–0.50)	0.30 (0.13–0.70)	<0.001 **	

* *p* < 0.05, ** *p* < 0.001.

## Data Availability

The raw data supporting the conclusions of this article will be made available by the authors, without undue reservations.

## Supplementary Materials

The following supporting information can be downloaded at: https://www.mdpi.com/article/10.3390/nu16183193/s1, Table S1: The subgroup analysis of fruit. Table S2: The subgroup analysis of root vegetables. File S1: The questionnaire for this study.
