# Supplementary material for: Correlation between Vegetable and Fruit Intake and Cognitive Function in Older Adults: A Cross-Sectional Study in Chongqing, China
Source: Nutrients. 2024 Sep 21;16(18):3193. doi: 10.3390/nu16183193 (PMC11435275; doi:10.3390/nu16183193)
Supplement: Supplementary file 1 [file nutrients-16-03193-s001.zip › File 2-questionare-t.pdf]

Annex II: Questionnaire

Questionnaire coding: surveyor:

Location: Survey time:

[introduction]

Dear Grandpa and grandma,

Hello, sincerely invite you to participate by the Chongqing medical university school of public health of "the elderly cognitive function and dietary factors relationship" survey, aims to understand the elderly cognitive function and diet behavior status, analyze by dietary improve the elderly cognitive function, delay the problems facing senile dementia, thus scientifically and effectively promote the elderly reasonable diet, prevent Alzheimer's disease, and improve the quality of life of the elderly.

This filling is anonymous form, all answers without right or wrong, please fill in according to your true situation; for all your answers, we are completely confidential; thank you very much for your participation and cooperation!

I have read this informed consent form, and I am the __________ for this survey

○ agreed to attend

○ Do not agree to participate (jump to the end of the questionnaire and answer it again)

I. Basic information

1、sex:

A.man B.woman

2、Age, and _______________

3、Your nation:

A .the Han nationality B. Other nationalities, the _________ ethnic

group

4、Your residence location is:

A. City (town) B.rural area

5、Do you live in a nursing home?

A.yes B.deny

6. At present, who takes main care of your daily life:

A .oneself B. Wife C. Children or their wives D.other person related by blood E. Nanny / caregiver F. Other, please indicate the ___________

7、 Your marital status is:

A.unmarried B.married C.divorce D.bereft of one's spouse

8、 Your educational level:

A. Never been to school B.primary school C.junior middle school D. High school / vocational high school / technical secondary school E.junior college F. Bachelor degree or above

9、 Industry / unit before your retirement:

A. Health care industry B. Education-related industries C. Government offices and public institutions (excluding medical and health care institutions and the education industry)

D . Business / Services E. Industry / Manufacturing F. Agriculture, forestry, animal husbandry and fishery G. Other ___________

10. Your current monthly income (including pension, pension, child support, etc.)?

A. Less than 1,000 yuan vs. B.1000-3000 yuan C.3001-5000 yuan D.5001-8000 yuan E. More than RMB 8,000 yuan

11. How often are you smoking?

A. Take more than 5 cigarettes a day, B. Draw less than 5 sticks per day to C. Greater than 1 per week, less than 1 per day

D. Less than one dose per week, E. Have quit smoking F. Never smoke

12. How often do you do physical activity every week?(Including doing housework, running, brisk walking, Taijiquan, ba Ba dance, etc.)

A.never B.1-2 times for the C.3-4 times D.5-6 times E. Admito 7 times

13. What is the average duration of each physical activity?

A .<0.5 hours for the B.0.5-1 hour C. At 1-2 hours D. Between 2-3 hours E.> 3 Hours

二、healthy condition

1. Height __________ cm (eg. 160.0cm)

2. Weight: __________ kg (e. g., 53.0kg)

3, the latest blood pressure measurement (systolic / diastolic blood pressure) ___________ / ___________ is not clear ()

4. Do you have any of the following diseases?

A.hypertension B.diabetes mellitus C.hyperlipemia D.cerebral apoplexy E.chronic obstructive pulmonary disease F.coronary disease G. Rheumatoid arthritis H.osteoporosis I.arthrolithiasis J. Other: ________ K.not have L. NK

5. How do you think of your current health status, compared to the rest of your peers?

A. Very bad B.difference C.secondary D.good E.fine F.hear nothing of

6. How much sleep do you usually get, including night and day?

A. Less than 6 hours vs. B. At 6-8 hours, C.8-10 hours D.10-12 hours E. More than 12 hours

三、Dietary and nutritional status survey

Food name		Number of meals taken (single choice)										
		Times / years			Time / month		Time / week			Time / day		
		0	1-2	3-6	1	2-3	1	2-3	4-6	1	2	≥3
1	Rice and its products (rice / rice flour / rice porridge, etc.)											
2	Wheat and its products (steamed bread / rolls / bread / biscuit, etc.)											
3	Coarse grains (brown rice / oat / buckwheat / barley, etc.)											
4	Potato type (potato / sweet potato, etc.)											
5	Beans (red beans / mung beans / soybean, etc.)											
6	Soybean products (tofu / dried bean / bean skin, etc.)											
7	Leaf vegetables (cabbage / lettuce / tail / lotus white, etc.)											
8	Solanum fruit (eggplant / tomato / green pepper, etc.)											
9	Root species (celery / lettuce / bamboo shoots / radish, etc.)											
10	Fruit category (apple / banana / kiwi, etc.)											
11	Meat (pork / beef / lamb, etc.)											

Food name		Number of meals taken (single choice)										
		Times / years			Time / month		Time / week			Time / day		
		0	1-2	3-6	1	2-3	1	2-3	4-6	1	2	≥3
12	Poultry meat (chicken / duck / goose meat, etc.)											
13	Pickled bacon											
14	Meat products (ham sausage / lunch meat, etc.)											
15	Visceral species (pig liver / pig heart / yellow throat / hairy belly / fat intestine, etc.)											
16	Bacteria and algae (mushrooms / kelp / seaweed, etc.)											
17	fish											
18	Other seafood (shrimp / crab / shellfish, etc.)											
19	Milk and its products (milk / yogurt, etc.)											
20	Eggs (eggs / duck eggs / quail eggs, etc.)											
21	Nuts (peanuts / seeds / walnuts / pistachio, etc.)											
22	beer											
23	spirit											
24	tea water											

4. Cognitive function survey

1. What is your memory?

A.beyond compare B.good C.same as D.mal E.very bad F.hear nothing of

2. Has your memory changed in the past year?

A. Get better. B.unchanged C. Worked worse D.hear nothing of

蒙特利尔认知评估基础量表中文版

Montreal Cognitive Assessment-Basic (MoCA-B) Chinese Version

姓名............................
性别..........　　年龄..........
教育年限........　　测试日期..................
检查者..........................

	得分
**执行功能**	开始时间 ______
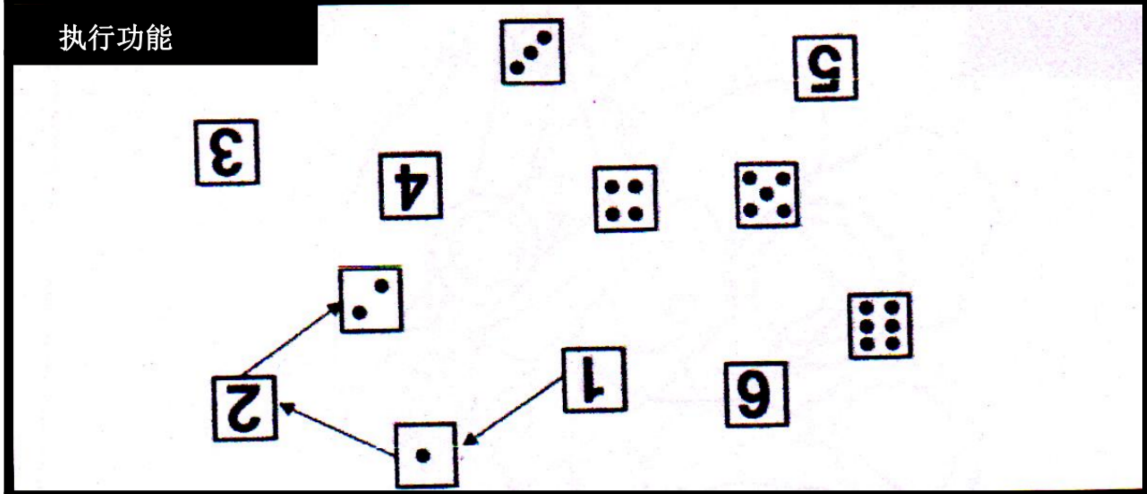	(/1)

即刻回忆

即使第一次测试所有词语均能回忆，也需完成第二次测试。

	梅花	萝卜	沙发	蓝色	筷子	不计分
第一次						
第二次						

流畅性　在 1 分钟内尽可能多的说出水果的名字　N=______个　(/2)

1-15 秒：	16-30 秒：	31-45 秒：	46-60 秒：	N≥13 计 2 分<br>N=8-12 计 1 分<br>N≤7 计 0 分

定向　[ ]时间(±2 小时)　[ ]星期几　[ ]月份　[ ]年份　[ ]地点　[ ]城市　(/6)

计算　用 1 元、5 元、10 元钱购买“13 元”的物品，说出 3 种付款方式。　(/3)

（说出 3 种正确付款方式计 3 分，2 种计 2 分，1 种计 1 分，未说出计 0 分）

正确方式：① ② ③ ④　　错误方式：______________________

抽象　下面的事物属于什么类别？（例如：香蕉-桔子=水果）　(/3)

[ ]火车・轮船　　[ ]锣鼓・笛子　　[ ]北方・南方

延迟回忆　(/5)

未经提示下自由回忆正确的词计分（每词 1 分）

	梅花	萝卜	沙发	蓝色	筷子
回忆时不提示	[ ]	[ ]	[ ]	[ ]	[ ]
分类提示	[ ]	[ ]	[ ]	[ ]	[ ]
多选提示	[ ]	[ ]	[ ]	[ ]	[ ]

视知觉　(/3)

图片识别，时间 60 秒。
图片见附录。

剪刀	T 恤	香蕉	台灯	蜡烛
手表	杯子	叶子	钥匙	勺子

N=9-10 计 3 分
N=6-8 计 2 分
N=4-5 计 1 分　(N=___)
N=0-3 计 0 分

命名　动物命名，图片见附录。　[ ]斑马　[ ]孔雀　[ ]老虎　[ ]蝴蝶　(/4)

注意

朗读**圆形**中的数字：
数列见附录　　1 5 8 3 9 2 0 3 9 4 0 2 1 6 8 7 4 6 7 5　　错误数___N
错误数≤1 个计 1 分　(/1)

朗读**圆形**和**正方形**中的数字：
数列见附录

3 8 5 1 3 0 2 9 2 0 4 9 7 8 6 1 5 7 6 4
1 5 8 3 9 2 0 3 9 4 0 2 1 6 8 7 4 6 7 5

错误数___N
错误数≤2 计 2 分
错误数=3 计 1 分
错误数≥4 计 0 分
(/2)

结束时间 ______

Adapted by : Qihao Guo MD　　Chinese version August 01,2015
Copyright : Z. Nasreddine MD　　www.mocatest.org

总分　(/30)

受教育年限<4 年加 1 分，不识字再加 1 分

总时间　　分　　秒

蒙特利尔认知评估基础量表中文版

Montreal Cognitive Assessment-Basic (MoCA-B) Chinese Version

附录

视知觉

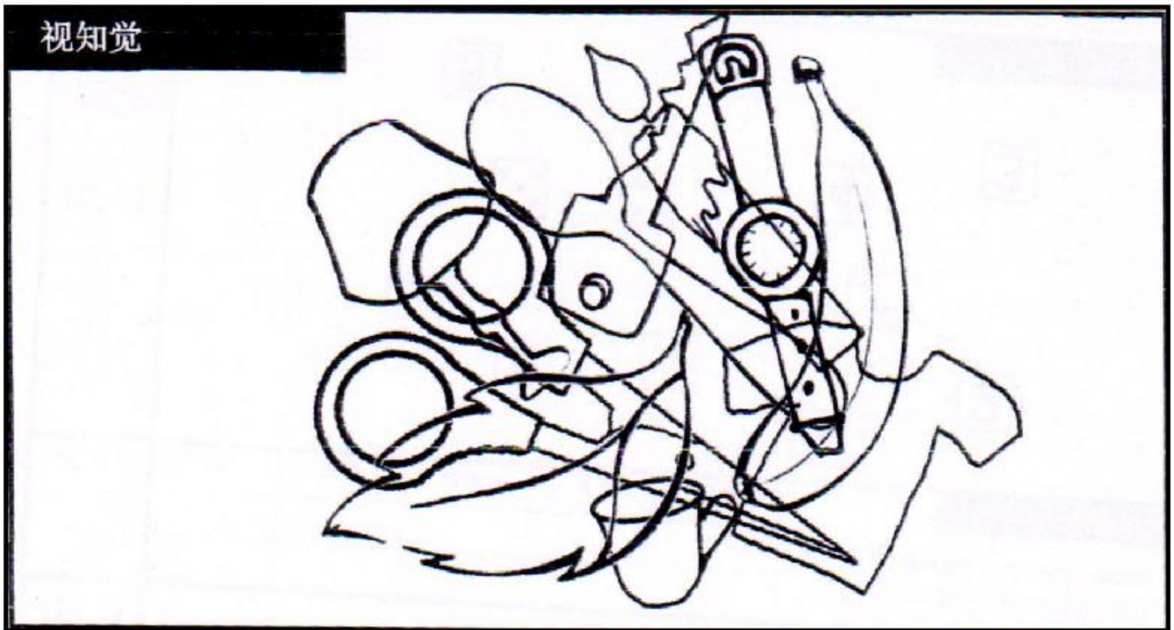

命名

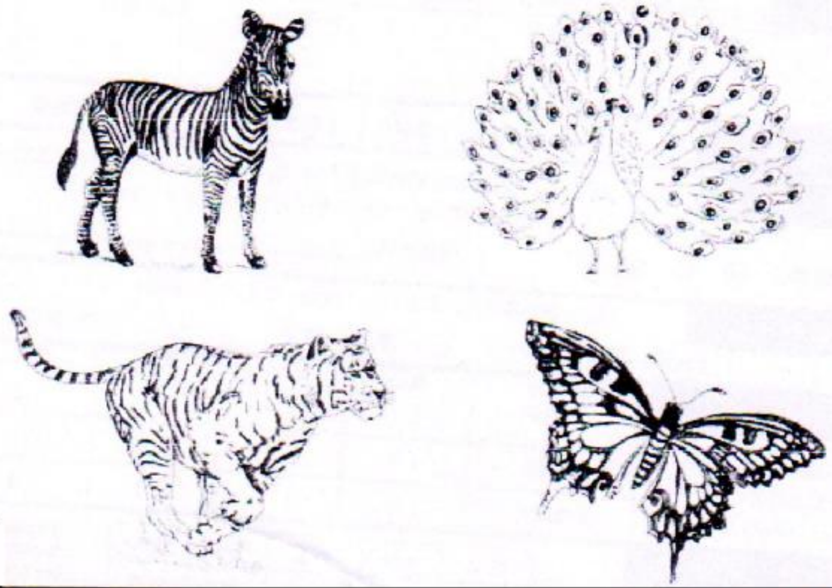

注意

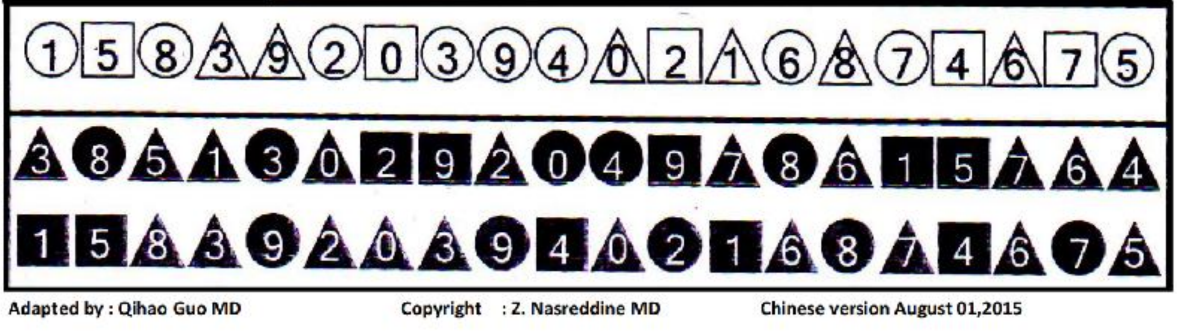

Adapted by : Qihao Guo MD

Copyright : Z. Nasreddine MD

Chinese version August 01,2015
