# Supplementary material for: Correlation between Vegetable and Fruit Intake and Cognitive Function in Older Adults: A Cross-Sectional Study in Chongqing, China
Source: Nutrients. 2024 Sep 21;16(18):3193. doi: 10.3390/nu16183193 (PMC11435275; doi:10.3390/nu16183193)
Supplement: Supplementary file 1 [file nutrients-16-03193-s001.zip › Table s1&s2-subgroup.pdf]

1. Subgroup analysis of the effects of fruit intake and socio-demographic associations on cognitive function scores

Table S1 indicated that there were interactions between BMI, smoking status and fruit intake (*p* for interaction<0.05), dose-response relationships were showed in every BMI groups and nonsmokers, however this relationship didn't exist among those smoked. Moreover, the effect of fruit intake on high and low cognitive function also had a dose-response relationship in specific populations. In concrete terms, those who were 60-79 years of age, male and female, married and other people, monthly income less than 3000 RMB and not engaged in mental labor prior to retirement, their results showed both higher levels (Q2, Q3 and Q4) of fruit intake was associated with higher scores on cognitive function (*p* for trend<0.05), whereas the above relationship was not presented in the rest of the population.

Table S1 Subgroup analysis of the effects of fruit intake and socio-demographic associations on cognitive function scores

	Intake of Fruit					
	Q1 (<21.43g/d)	Q2 (21.43~53.57 g/d)	Q3 (53.57~115 g/d)	Q4 (⩾115 g/d)	*p* for trend	*p* for interaction
Age						0.534
60-79	ref	0.53 (0.33-0.85)	0.40 (0.26-0.62)	0.34 (0.21-0.56)	<0.001**	
⩾80	ref	0.32 (0.09-1.22)	0.30 (0.07-1.18)	1.11(0.10-12.31)	0.376	
Sex						0.061
Male	ref	0.68 (0.36-1.30)	0.73 (0.37-1.43)	0.39 (0.21-0.72)	0.004*	
Female	ref	0.48 (0.27-0.85)	0.27 (0.16-0.45)	0.50 (0.22-1.15)	<0.001**	
BMI						0.032*
Normal weight	ref	0.73 (0.44-1.21)	0.55 (0.34-0.89)	0.49 (0.28-0.84)	0.004*	
Underweight	ref	0.13 (0.05-0.39)	0.16 (0.06-0.43)	0.16 (0.03-0.73)	<0.001**	

Overweight /Obesity	ref	1.57 (0.42-5.90)	0.38 (0.11-1.23)	0.27 (0.07-0.98)	0.011*	
Marital status						0.233
Married	ref	0.65 (0.40-1.06)	0.52 (0.33-0.83)	0.37 (0.22-0.64)	<0.001**	
Other	ref	0.43 (0.18-1.05)	0.20 (0.09-0.45)	0.31 (0.12-0.82)	<0.001**	
Pre-retirement job						0.330
Mental labor	ref	0.93 (0.29-2.94)	0.42 (0.13-1.33)	0.64 (0.20-2.12)	0.230	
Physical labor	ref	0.62 (0.37-1.06)	0.57 (0.35-0.93)	0.35 (0.19-0.62)	<0.001**	
Other	ref	0.35 (0.13-0.94)	0.18 (0.07-0.48)	0.20 (0.06-0.68)	<0.001**	
Average monthly income						0.114
<1000 RMB	ref	0.73 (0.35-1.56)	0.24 (0.11-0.53)	0.16 (0.06-0.40)	<0.001**	
1000 RMB–3000 RMB	ref	0.54 (0.30-1.00)	0.59 (0.34-1.01)	0.47 (0.24-0.94)	0.027*	
>3000 RMB	ref	0.79 (0.25-2.50)	0.49 (0.16-1.51)	0.69 (0.23-2.11)	0.443	
Smoking						0.043*
Non-smoker	ref	0.64 (0.40-1.04)	0.34 (0.21-0.53)	0.31 (0.17-0.58)	<0.001**	
Smoker	ref	0.19 (0.06-0.63)	0.66 (0.27-1.61)	0.47 (0.22-1.02)	0.166	

*$p<0.05$, **$p<0.001$

2. Subgroup analysis of the effects of root vegetables intake and socio-demographic associations on cognitive function scores

Table S2 indicated that there were interactions between sex, smoking status and root vegetables intake (p for interaction<0.05), and no matter if they were or not smoked, the dose-response relationships existed, so did the sex groups. Moreover, the effect of root

vegetables intake on high and low cognitive function also had a dose-response relationship in specific populations. For instance, people aged 60-79 year old, with normal and underweight BMI, married, monthly income less than 3000 RMB and engaged in physical labor, their results showed both higher levels (Q2, Q3 and Q4) of root vegetables intake were proved to be a protective factor for cognitive impairment (p for trend<0.05), whereas the above relationship was not presented in the rest of the population.

Table S2 Subgroup analysis of the effects of root vegetables intake and socio-demographic associations on cognitive function scores

	Intake of Root vegetables					
	Q1 (<14.64g/d)	Q2 (14.64~25.71g/d)	Q3 (25.71~82.14g/d)	Q4 (⩾ 82.14g/d)	*p* for trend	*p* for interaction
Age						0.636
60-79	ref	0.48 (0.30-0.76)	0.41 (0.26-0.64)	0.41 (0.25-0.66)	<0.001**	
⩾80	ref	0.99 (0.27-3.70)	0.84 (0.25-2.80)	0.79 (0.18-3.39)	0.700	
Sex						<0.001**
Male	ref	0.76 (0.36-1.61)	0.31 (0.16-0.62)	0.57 (0.27-1.16)	0.024*	
Female	ref	0.39 (0.23-0.66)	0.87 (0.49-1.56)	0.31 (0.16-0.58)	0.003*	
BMI						0.561
Normal weight	ref	0.56 (0.33-0.94)	0.59 (0.36-0.96)	0.43 (0.25-0.75)	0.005*	
Under weight	ref	0.43 (0.16-1.11)	0.23 (0.08-0.66)	0.47 (0.15-1.41)	0.037*	
Overweight /Obesity	ref	0.38 (0.11-1.26)	0.22 (0.07-0.74)	0.39 (0.11-1.38)	0.064	
Marital status						0.265

Married	ref	0.51 (0.31-0.83)	0.37 (0.22-0.60)	0.44 (0.26-0.74)	<0.001**	
Other	ref	0.47 (0.20-1.09)	0.73 (0.32-1.66)	0.33 (0.13-0.84)	0.053	
Pre-retirement job						0.096
Mental labor	ref	0.67 (0.23-1.99)	1.12 (0.38-3.27)	1.31 (0.43-3.96)	0.401	
Physical labor	ref	0.54 (0.31-0.93)	0.31 (0.18-0.52)	0.29 (0.16-0.51)	<0.001**	
Other	ref	0.45 (0.16-1.24)	0.63 (0.25-1.61)	0.46 (0.15-1.46)	0.275	
Average monthly income						0.168
<1000 RMB	ref	0.48 (0.22-1.01)	0.33 (0.16-0.67)	0.14 (0.05-0.41)	<0.001**	
1000 RMB–3000 RMB	ref	0.45 (0.24-0.84)	0.47 (0.26-0.85)	0.48 (0.26-0.88)	0.020*	
>3000 RMB	ref	1.22 (0.40-3.66)	0.79 (0.24-2.59)	1.33 (0.42-4.20)	0.830	
Smoking						0.003*
Non-smoker	ref	0.48 (0.30-0.76)	0.68 (0.43-1.09)	0.40 (0.24-0.68)	0.003*	
Smoker	ref	0.53 (0.19-1.46)	0.14 (0.05-0.38)	0.40 (0.15-1.06)	0.018*	

$^{*}p<0.05$, $^{**}p<0.001$
